# Factors associated with obstetric fistulae occurrence among patients attending selected hospitals in Kenya, 2010: a case control study

**DOI:** 10.1186/1471-2393-13-56

**Published:** 2013-02-28

**Authors:** Zeinab Gura Roka, Mathias Akech, Peter Wanzala, Jared Omolo, Sheba Gitta, Peter Waiswa

**Affiliations:** 1 Kenya Field epidemiology and laboratory program Ministry of Public Health and Sanitation Kenya, P.O. BOX 21691–00100, Nairobi, Kenya; 2 Jomo Kenyatta University of Agriculture and Technology, Juja, Kenya; 3 Centre for Public Health Research, Kenya Medical Research Institute, Nairobi, Kenya; 4 Kenya Ministry of Public Health and Sanitation, Nairobi, Kenya; 5 African Field Epidemiology Network, Kampala, Uganda; 6 Makerere University School of Public Health, College of health Sciences, Kampala, Uganda; 7 Division of Public Health, Division of Global Health, Karolinska Institutet, Stockholm, Sweden

**Keywords:** Obstetric fistula, Vesico vaginal fistula, Fistula

## Abstract

**Background:**

In Kenya, about 3000 fistula cases are estimated to occur every year with an incidence of 1/1000 women. This study sought to identify risk factors associated with developing obstetrics fistula in order to guide implementation of appropriate interventions.

**Methods:**

An unmatched case control study was conducted in three major hospitals in Kenya between October and December 2010. Cases were patients who had fistula following delivery within the previous five years. Controls were systematically selected from women who attended obstetrics and gynecology clinics at these hospitals, and did not have present or past history of fistula. Odds ratio was used as measure of association with their corresponding 95% confidence interval. Factors with p value of <0.1 were included into forward additive logistic regression model to generate adjusted odds ratios.

**Results:**

Seventy cases and 140 controls were included in the study. Independent risk factors associated with obstetrics fistula included duration of labour of >24 hours (OR = 4.7, 95% CI = 2.4 -9.2), seeking delivery services after 6 hours of labour onset (OR = 6.9, 95% CI = 2.2-21.3), taking more than 2 hours to reach a health facility (OR = 5.7, 95% CI = 2.9 -11.5), having none or primary education (OR = 9.6, 95% CI = 3.3 –27.9) and being referred to another facility for emergency obstetrics services (OR = 8.6, 95% CI = 2.7 –27).

**Conclusions:**

Risk factors for developing obstetrics fistula were delays in care seeking including delay in making decision to seek delivery servers after six hours of labour onset, taking more than two hours to reach a health facility, labour duration of more than 24 hours and having no formal or primary education. Efforts geared at strengthening all levels of the health system to reduce delays in access to emergency obstetric care are needed.

## Background

Obstetrics fistulae are abnormal communications created between the vaginal wall and the bladder (vesico-vaginal fistula) and or the rectum (recto vaginal fistula). Some women with obstretric fistula can have near miss morbidity and World Health Organisation (WHO) referred to fistulae as the single most devastating morbidity of neglected child birth [1]. It is predominantly caused by a prolonged and or obstructed labour in Sub-Saharan Africa; however, abdominal hysterectomy remains the most common cause of vaginal fistula in developed countries [2]. In Egypt, 98% of the fistulae are iatrogenic following surgeries and only two% are caused by obstructed labour [3].

The WHO estimates that approximately two million women have untreated obstetrics fistula with a worldwide incidence of 1–2 per 1 000 deliveries; majority living in Sub-Saharan Africa [4]. Obstetric fistula was a global problem, however it was eradicated in Europe and North America following improved obstetric care but the condition remains prevalent in Sub-Saharan Africa and Asia. In Africa, most studies on fistula are hospital based and report incidences ranging between 0.6 and 3.5 /1,000 deliveries [5-7]. The estimated national prevalence of obstetric fistula in Ethiopia is 1% of ever married women [8]. In Kenya, it is estimated that annually there are 3,000 new fistulae cases but only 7.5% are reported and treated [9].

Obstetrics fistulae result in physical, psychological and social effects. The continuous leakage of urine and or feaces causes a bad odour which may lead to isolation, stigmatization, rejection by society and divorce [10]. Many of these women also do not engage in any economically gainful activities thus becoming an economic burden to society [11].

Major risk factors for obstetrics fistulae include early age at pregnancy, short stature, illiteracy, poverty, not attending antenatal care and rural place of residence or living far away (>3 km) from a health facility [10,12,13]. According to the delay model discussed by Thaddeus and Maine, there are three delays that contribute to obstetrics fistula development; delay in the making the decision to seek care, delay in arrival at a health facility; and delay in the provision of adequate care [14]. Most studies have looked at the total duration of labour as opposed to the duration that lapsed between onset of labour and the decision to seek delivery services and or reaching health facilities. Some risk factors are unique to specific regions and countries for example the ‘gishiri” cut practiced in northern Nigeria [12,15]. Previous evaluations of obstetric fistula in Kenya have all been descriptive in nature. The need to carry out analytical studies to identify risk factors so as to focus the existing interventions has largely remained unfulfilled. We therefore conducted an unmatched case control study to identify the factors associated with obstetrics fistula among patients attending selected hospitals in Kenya.

## Methods

### Study design and sites

An unmatched case–control study was conducted between October and December 2010 at three selected hospitals in Kenya namely Kenyatta National Hospital in Nairobi, Coast Provincial General Hospital in Mombasa and New Nyanza Provincial Hospital in Kisumu. In Kenya, apart from the Kenyatta National Hospital and Moi Teaching and Referral Hospital, most of the fistula surgeries are done as yearly surgical camps. These camps are mainly held in these three hospitals. Kenyatta National Hospital serves as the referral point for fistula management especially for complicated and complex fistulae. The fistula clinic at Kenyatta National Hospital has been operating since 1994.In 2009, fistula surgeries have been subsidized courtesy to collaboration with Africa Medical Research Foundation (AMREF). In this clinic, 10–15 patients with fistula are seen every Tuesday and appropriate management planned for them. The other two hospitals are provincial referral hospitals and sites for yearly vesico-vaginal fistula repair. The Kenya health system has six levels of public health facilities. The first level is community units which are manned by community health workers. Level two are the dispensaries which offer outpatient services for common ailments and also provide basic primary health services like immunization. Level three are the health centres which offer outpatient, delivery of normal labour and immunization but lack the capacity to offer emergency obstetrics services like caesarean section. Level four are the district hospitals, level five are provincial hospitals and level six are the Kenyatta National Referral Hospital and Moi Teaching and Referral Hospital. Levels four, five and six can offer the comprehensive obstetrics care and all patients with complicated labour are referred to these facilities***.***

According to Kenya Demographic Health Survey, 2010, it is estimated that 92% of pregnant women attend antenatal care at least once in their pregnancy but only 43% deliver in health facilities.

### Study population and inclusion criteria

The Study population included women who had delivered within the previous five years attending the three study sites. A case was defined as a woman with treated or untreated fistula following pregnancy within the previous five years at the gynecological clinic at the study sites and had consented to take part in the study. The five year limit was necessary to minimise recall bias of events surrounding the delivery preceding the fistula development. A control was defined as a woman who had delivered within the previous five years no history of treated or healed fistula, in obstetrics and gynecology clinic at the study sites and had consented to take part in the study. Women who were nulli-parous were excluded from the study.

### Sample size and sampling

A minimum sample size of 207 using Fleiss (1981) formulae was calculated with the case to control ratio of 1:2, 95% confidence interval and power at 80% to detect a minimum odds ratio of two. Cases were selected from the study sites proportionate to the number of cases seen at the site in 2009. Case-patients who met the inclusion criteria were enrolled consecutively as they came to the clinic until the desired sample size was achieved.

Number of controls was double the number of cases selected from each study site. The number of controls recruited per day was obtained by dividing number of total controls to be recruited from a study site by the number of clinic days within the study period. Every day the files of the patient were reviewed to select the ones who satisfied the criteria for being a control who were then selected using simple random sampling with replacement.

### Data collection, management and analysis

Data was collected using a semi-structured interviewer administered questionnaire via face-to-face interviews. The data included socio demographic variables, circumstances surrounding most recent delivery which included duration of labour and duration before going to a health facility among others and clinical information which was collected from cases.

Descriptive analyses of the demographic variables for both cases and controls were done using means, proportions and frequencies. Crude odds ratios and 95% confidence intervals were calculated at bivariate analysis where exposures were tested for association with the outcome variable (obstetrics fistulae) using chi-square test or Fisher’s exact test. Adjusted odds ratio and their 95% confident interval were calculated by including all exposures with p value <0.1 in the multivariate model. All exposure with P value < 0.05 were considered statistically significant.

### Ethical considerations

Ethical review and approval was sought and obtained from Scientific Steering Committee (SSC) and Ethical Review Committee (ERC) of Kenya Medical Research Institute, SSC no. 1881. Clearances to carry out the study were sought and obtained from the hospitals where studies were carried out (Kenyatta National Hospital, Coast Provincial General Hospital and Nyanza Provincial General Hospital). Written informed consent was sought and obtained from each participant.

## Results

Seventy cases and 140 controls were included into the analysis. Majority of the case were aged 15-24 (55%, 39/70) while controls were aged 25–34 years (59%, 82/140). The median age at first pregnancy was 22.5 (IQR 11) years for cases and 29 (IQR 9) years for controls (Table 1). Seventeen percent of the cases (12/70) had their first pregnancy when they were 11–15 years old and 64% (45/70) had their first pregnancy by the time they were 20 years old. Thirty-nine percent (54/140) of the controls had their pregnancy by the time they were 20years old (Table 2). Fifty percent (35/70) of the cases were primi-parous and number of cases reduced as parity increased.

**Table 1:** Socio-demographic characteristics for cases and controls attending Coast Provincial, Kenyatta National and Nyanza Provincial hospitals in Kenya, October to December 2010

**Characteristics**	**Cases (n = 70) Freq (%)**	**Controls (n = 140) Freq (%)**	*P-value
Median age at first pregnancy	22.5 (IQR11)	29 (IQR9)	<0.01
Marital status			<0.01
Married	39(55.0)	110(78.6)	
Single	12(17.0)	19(14.3)	
Divorced	14(20.0)	6(4.3)	
Widowed	4(7.0)	5(2.9)	
Parity			<0.01
1	35(50.0)	35(25.0)	
2	14(20.0)	39(27.9)	
≥3	21(30.0)	66(47.1)	
Height			<0.01
≤150cm	31(44.3)	0(0.0)	
>150cm	39(55.7)	140(100.0)	
Level of education			<0.01
None	23(32.8)	4(2.9)	
primary	34(48.4)	42(30.0)	
Secondary	11(16.9)	52(37.1)	
Tertiary	2(3.0)	42(30.0)	

*P-value derived from chi square and fishers test.

**Table 2:** Obstetrics characteristics of cases and controls attending Coast Provincial, Kenyatta National and Nyanza Provincial hospitals in Kenya, October to December 2010

**Variables**	**Cases**	**Controls**
	**Freq (%)**	**Freq (%)**
Duration of labour	n = 70	n = 140
≤12	0(0.0)	64(45.7)
13-24	3(4.3)	54(38.6)
25-48	9(12.9)	21(15)
49-72	23(32.9)	1(0.0)
>72	35(50)	0 (0.7)
Place of delivery	n = 70	n = 140
Home	19(27.1)	22(15.7)
Health Facility	51 (72.9)	118(84.3
Duration from onset of labour to decision to seek services	n = 51	n = 118
≤6 hours	9 (17.6)	72(61.0)
>6 hours	42 (82.4)	46(39.0)
Time taken from home to reach the a h/facility	n = 51	n = 118
≤2 hours	13 (25.5)	94(79.7)
>2 hours	38 (74.5)	24(20.3)
Referral to another facility	n = 51	n = 118
Yes	28 (54.9)	12(10.1)
No	25 (45.1)	108(89.9)
Mode of delivery	n = 70	n = 140
Caeserian section	42(60)	27(19.3)
Spontaneous vaginal delivery	23(32.9)	109(77.9)
Vacuum delivery	4(5.7)	4(2.9)
Forceps delivery	1(1.4)	0(0.0)
Delivery outcome	n = 70	n = 140
Live birth	15(21.4)	135(96.4)
Still birth	55(78.6)	5(3.6)
Antenatal attendance	n = 70	n = 140
No	18(25.7)	9 (6.4)
1	10(19.2)	2(1.4)
2	11(21.2)	12(8.6)
3	11(21.2)	37(26.4)
4+	20(38.4)	80(57.2)
Age group at marriage	n = 58	n = 123
<15	16(27.6)	5(4.1)
16-20	29 (50.0)	51(41.5)
21-25	10 (17.2)	47(38.2)
26-30	2 (3.4)	17 (13.8)
31-35	0 (0.0)	3(2.4)
36-40	1( 1.7)	0 (0.0)
41-45	1(1.7)	0(0.0)
**Age group at first childbirth**	**n = 70**	**n = 140**
11-15	12( 17.1)	4(2.9)
16 - 20	33 (47.1)	50 ( 35.7)
21 - 25	19 (27.1)	54 (38.6)
26 - 30	4 (5.7)	25 (17.9)
31 - 35	1(1.4)	5 (3.6)
36 - 40	1 (1.4)	2 (1.4)

Comparing the cases and controls, the mean age at first pregnancy was higher in controls than in cases (p < 0.01). Cases had mostly none (33%, 23/70) or primary education (48%, 34/70) while most controls (67%, 94/140) had secondary and tertiary education (p value < 0.01) (Table 1). Of the 70 cases, 38(54%) were married and 14(20%) were divorced compared to controls where 110/140 (79%) were married and 6/140(4.3%) were divorced. All the women with fistula who were divorced attributed the divorce to the fistula onset. Only 3% (2/70) of the cases had formal salaried employment compared to controls where 16% (23/140) had formal salaried employment. Thirty-four percent of cases report to have stopped working after onset of symptoms of fistula. Almost half of the cases (44%, 31/70) were shorter than 150cm while all controls were taller than 150 cm.

As shown in Table 2, half of the cases (35/70) reported a labour duration of more than 72 hours. Forty six percent (64/140) of the controls had labour duration of less than 12 hours and 39% (54/140) had duration of 12–24 hours. Seventy three percent (51/70) of cases and 84% (118/120) of controls delivered in a health facility. Eighty-two percent (42/51) of the cases made the decision to seek delivery services after six hours of labour had elapsed while 61% (72/118) of controls made the decision within six hours of labour. Seventy five percent (38/70) of the cases took more than two hours to get to a facility irrespective of mode of transport while 80% (94/118) of controls took two hours or less to get to a health facility. Seventy four percent (52/70) of cases and 94% (121/140) of controls had attended antenatal clinic at least once in the last pregnancy or the pregnancy associated with fistula.

Eighty six percent of the cases who were primipara were younger than 20 years while only 20% of the women with parity of more than one were younger than 20 years (Table 3).

**Table 3:** Distribution of fistula cases attending Coast Provincial, Kenyatta National and Nyanza Provincial hospitals in Kenya, October to December 2010 by age-group and parity

**Age-group**	**Parity**	
	**Primipara**	**>1 parity**
11-15	10(28.6)	2(5.7)
16-20	20(57.1))	5(14.3)
21-25	5(14.3)	3(8.6)
26-30	0(0.0)	12(34.3)
31-35	0(0.0)	5(14.3)
36-40	0(0.0)	6(17.1)
41-45	0(0.0)	2(5.7)

At multivariate analysis (Table 4), independent risk factors associated with obstetrics fistula included duration of labour of >24 hours (OR = 4.7, 95% CI = 2.4 -9.2), making the decision to seek delivery services after more than 6 hours of labour at home (OR = 6.9, 95% CI = 2.2-21.3), taking more than two hours to reach a health facility (OR = 5.7, 95% CI = 2.9 -11.5), having none or primary education (OR = 9.6, 95% CI = 3.3 –27.9) and being referred to another facility for emergency obstetrics services (OR = 8.6, 95% CI = 2.7 –27).

**Table 4:** Factors associated with occurrence of obstetrics fistula among cases and controls attending Coast Provincial, Kenyatta National and Nyanza Provincial hospitals in Kenya, October to December 2010

**Variables**	**Cases freq (%)**	**Control freq (%)**	**OR**	**95% CI**	**aOR**	**95% CI**
Level of education Secondary + =1.00	57/70(81.4)	46/140(32.8)	8.9	4.3-19.4	9.6	3.3-27.9*
Referred to another facility(Yes/NO)	23/51(45.1)	10/118(8.5)	8.9	3.5-22.8	8.6	2.7 -27.0*
Duration of labour before deciding to seek delivery services >6hrs/<6	42/51(82.3)	48/118(51.7)	6.8	2.9-16.6	6.9	2.2 -21.3*
Time taken to reach the h/facility (>2hrs/<2hrs)	38/51(74.5)	24/118(20.3)	11.5	4.9-26.9	5.7	2.9 -11.5*
Labour duration >24hrs/<24	67/70(95.7)	21/140(15)	119	32-520	4.7	2.4-9.2*
Age at marriage >18/≤18yrs	30/57(52.6)	95/120(79.3)	0.3	0.1-0.6	0.2	0.03-1.1
Age at first child >18/≤18yrs	30/70(42.9)	117/140(83.6)	0.15	0.07-0.3	0.4	0.08-2.3
Antenatal clinic attendance(Yes/No)	52/70(74.3)	131/140(93.6)	0.2	0.08-0.5	0.13	0.006-2.4
Formal occupation (Yes/No)	2/70(2.9)	23/140(16.4)	0.2	0.02 -0.6	0.4	0.05-1.3
Antenatal visit (>1/1)	42/52(80.8)	129/131(98.5)	0.07	0.01-0.3	0.1	0.03-4.6
Primi parity	35/70(50)	36/140(25)	2.9	1.5-5.5	1.5	0.36-6.9

*Significant at Multivariate analysis.

## Discussion

In this unmatched case control study, we examined factors associated with obstetrics fistulae in three selected hospitals in Kenya. Our analysis identified having none or primary education, making decision to seek delivery services after six hours from onset of labour, and reaching a health facility after two hours as risk factors for developing obstetrics fistula. Other risk factors included delay caused by referral to another facility and duration of labour of more than 24 hours. This is one of the first analytical studies to evaluate obstetric fistula in Kenya. Previous studies been mainly descriptive thus limiting the understanding of risk factors.

Similar case control studies carried out in Nigeria [10,13] identified low levels of education and labour duration of more than 24 hours as risk factors for obstetrics fistula occurrence. However, the study did not evaluate the delay in making the decision to seek delivery services and in reaching health facilities. In a descriptive study from Zambia [16], women were asked where they delayed and 67.5% said they delayed at home. This delay at home was not evaluated for association with fistula occurrence. It is important for a woman to be in a health facility once she is in active labour in order to get proper management and good outcome. Cultural beliefs, economic constrains and attitude towards services in health facilities could contribute to this delay [17]. Attendance by unskilled persons who did not recognise impending complications early enough to seek emergency obstetrics services could also contribute to delay in seeking delivery services [17]. Increasing awareness and importance of timely health facility delivery and dangers signs in pregnancy among pregnant mothers and the community is important in reducing this delay. Community health workers could be used to raise this awareness.

In this study we considered the duration of time they took to reach the health facilities irrespective of mode of transport. Taking more than two hours to reach a health facility was a significant risk factor for developing obstetric fistula. Findings by Melah from Nigeria [10] show that distance of more than 3km from the women’s homes to the health facilities is a risk factor for obstetrics fistula. Transport problems and long distances have been identified as contributors to delay in reaching the health facilities [16,17]. Maternal waiting shelters for pregnant women who live long distances from the hospitals to stay in when they reach near term or expected dates of delivery, will help in reducing this delay in the short term [16]. In the long term, access to functional hospitals needs to be improved through better transportation or distribution of health facilities. Communities could also initiate low cost transportation system which will enable the women to get to the health facilities in time.

Primary health care facilities in Kenya do not offer emergency obstetrics services for women with obstructed labour and therefore they have to be referred to district hospitals or higher levels for possible caesarean section and other management. The duration taken to get to the referral facility adds to delay in getting appropriate management and this is specially so when coupled with delay in seeking delivery services and delay in reaching a health facility. Thus there is urgent need to build the capacity of primary level health care to conduct emergency obstetrics care and also to improve referrals between health facilities.

In this study difference in antenatal attendance among cases and controls was not significant. Findings in studies from Nigeria [10,12,13,16], indicate that not attending antenatal care was a significant risk factor for developing fistula. This non-significant finding could be explained by the increased advocacy for antenatal care attendance in Kenya where women are asked to present their antenatal cards when they take their children for immunization. Antenatal attendance was relatively high among cases but women still delayed in making the decision to seek delivery services. There is therefore need to improve on quality of the antenatal services to increase awareness of importance of timely hospital delivery.

Compared to controls, cases were shorter (44.3% <150 cm), had no formal employment, had lower antenatal attendance and had either no formal education or only reached the primary school level. These findings are comparable to other studies in Africa [12,16,18]. Mean age at first pregnancy among cases in this study was higher than in most studies in Africa, [16,19,20] however; the difference between cases and controls was not significant as an independent risk factor. This could mean that the fistula cases were not due to mainly an underdeveloped pelvis and highlights the importance of timely appropriate delivery services for complicated deliveries.

Half of the cases in this study had parity of two and above. This is consistent with findings by Holme from Zambia [16]. Most of the multi-parous women had delivered at home in earlier pregnancies and did not realize the need of timely health facility delivery. As we plan preventive measures, we need to take this into account and tailor our interventions to cover multi-parous women.

In this study, almost a quarter of the cases were divorced and all of them attributed the divorce to fistula development. This proportion is lower than figures in other studies from Ethiopia and Nigeria [12,19,21]. The lower proportion in this study could be explained by the fact that polygamy is practised within most communities without actually divorcing the other woman. In Zambia, the proportion of divorced women with fistula is similar to findings in this study [16]. Being divorced after onset of fistula together with the fact that 34% of cases report to have stopped working after onset of symptoms of fistulas how the social effect of obstetrics fistula among women.

### Methodological considerations

In this study, the controls were all taller than 150 cm and thus this variable could not be added to the model to evaluate for its association with obstetrics fistulae occurrence. The data collected were mainly self reported by the participants and specifically the data on circumstances surrounding delivery could not be verified from partographs which are usually kept at facilities they delivered in. Recall bias was minimized by including women who delivered within the previous five years. The retrospective recall of labor and transportation time makes the time only approximate. In future studies, there is a need to separate fistulae occurring in the first versus subsequent pregnancies because while their causes may overlap, there is likely to be distinct factors in the first pregnancy (small pelvis) that differ with fistulae formation in subsequent pregnancies (malposition). This study doesn’t give indications on magnitude of obstetrics fistula due to limitation of the study design and it is also hospital based.

## Conclusions

From this study, the significant risk factors associated with developing fistula among women attending the three selected hospitals in Kenya are mainly delays in getting or accessing emergency obstetrics services and illiteracy among women.

Addressing the causes of these delays and increasing timely access to emergency obstetrics services will reduce the magnitude of obstetrics fistula. Quality antenatal care and educating pregnancy women on importance of timely hospital delivery will reduce the delay in seeking delivery services. Availability of emergency obstetrics services (caesarean sections) at selected primary health care facilities whereby selected health centres are equipped with maternity theatres and other surgical equipments in order to reduce the delay caused by referrals of cases to other facilities. In the long run, infrastructure improvement, poverty reduction and high level of literacy among the women will reduce the delays in accessing delivery services.

## Abbreviations

WHO: World health organization; OR: Odds Ratio; ERC: Ethical review committee; SSC: Scientific steering committee; CI: Confidence interval; KFELTP: Kenya field epidemiology and laboratory training program; CDC: Centres for disease control and prevention; AFENET: African field epidemiology network; AMREF: Africa medical reaserch foundation.

## Competing interests

The authors declare that they have no competing interests.

## Authors’ contributions

ZG is the principal investigator and contributed to developed of the concept, collected data, analyzed data and wrote the draft and final article. JO contributed to development of concept, data collection, data analysis and reviewed the draft and final article. MA contributed to development of concept, collection of data and analysis. PW1 contributed to development of concept, collection of data and analysis. PW2 critically reviewed the draft and final article. SG critically reviewed the draft and final article. All authors read and approved the final manuscript.

## Pre-publication history

The pre-publication history for this paper can be accessed here:

http://www.biomedcentral.com/1471-2393/13/56/prepub
